# Are board games useful for people with dementia? A preliminary study for a non-pharmacological intervention

**DOI:** 10.3389/fpsyg.2025.1569406

**Published:** 2025-09-18

**Authors:** Veronica Guardabassi, Alessandro Maranesi, Marta Di Massimo, Evelyn Manoni, Elisa Cirilli, Paola Nicolini

**Affiliations:** Department of Humanities, University of Macerata, Macerata, Italy

**Keywords:** dementia, board games, cognitive functioning, well-being, psycho-social intervention, non-pharmacological intervention

## Abstract

**Introduction:**

The diagnosis of dementia presents a serious public health concern and a challenge for the entire scientific community. Among the potential lines of intervention, board games can be considered a non-pharmacological strategy. This study aimed to explore the potential of board games as a group-based intervention to support cognitive stimulation and psychological well-being in people with dementia.

**Methods:**

This study included 36 older people diagnosed with dementia (*M*_age_ = 86.13; *SD* = 6.94; *M*_MMSE_ = 18.82, *SD* = 2.96). Participants performed verbal working memory, verbal fluency and denomination tasks at the beginning of the project (T1), played with board games with their facilities’ professionals over a month (S1), and, at the end, they did a parallel form of the initial cognitive tasks (T2). Then, they played with their games’ conductors for a further 2 months (S2), and at the end of this period (T3), they performed the initial cognitive tasks. The research team observed participants’ level of well-being during S1, and facility professionals completed a questionnaire at T1, T2, and T3 to provide information about participants’ symptoms of depression (T1 and T3) and their point of view on board game activity (T2 and T3).

**Results:**

The results showed that cognitive functions do not increase over time [*F* (2, 32) = 2.54, *p* = 0.095, *η*^2^ = 0.145]. However, there was a significant change after the first month (*p* = 0.029). Specifically, the verbal fluency improved from T1 to T2 (*p* = 0.042). In addition, the results indicated that the level of well-being observed during board game activities was higher than the level of discomfort [*F* (1, 35) = 133.69, *p* > 0.001, *η*^2^ = 0.876]. Professionals’ responses to open-ended questions about cognitive functioning and psychological well-being corroborated the findings. No changes in depression symptoms were found [*F* (3, 19) = 2.39, *p* = 0.105, *η*^2^ = 0.297].

**Discussion:**

The results of this study suggest that board games can be explored further as a non-pharmacological intervention for groups of older adults diagnosed with dementia, as favorable outcomes were observed in terms of cognition and well-being. Theoretical implications, research directions, and professional perspectives were also discussed.

## 1 Introduction

The World Health Organization (WHO, 2023) estimates that by 2050, approximately 2.1 billion people will be over the age of 60 and that the number of individuals aged 80 and above will triple during the same period. These data have drawn increased attention to the health and social issues affecting the older population (Cammisuli et al., 2022). A common phenomenon associated with aging is the decline in cognitive functions. The increase in neurodegenerative diseases, such as dementia, represents a serious global public health issue (Nemeth et al., 2017; D’Cunha et al., 2019; Irazoki et al., 2020) and a significant challenge for the entire scientific community (Sykora et al., 2015; Gómez-Soria et al., 2023a, 2023b).

### 1.1 Dementia syndromes

Dementia syndromes comprise a collection of signs and symptoms characterized by a progressive decline in cognitive abilities or neuropsychiatric behavioral changes. These changes can lead to a loss of cognitive and motor functions in older individuals, affecting their ability in performing daily activities (Clare et al., 2003; Nascimento et al., 2021; Gómez-Soria et al., 2023a, 2023b). It can be said that dementia is an umbrella term for a range of neurodegenerative disorders that impair cognitive functions such as memory, language, and purposeful actions, thereby reducing the quality of life (D’Cunha et al., 2019). Memory difficulties, in particular, can have a significant impact on self-confidence and may lead to anxiety, depression (Watt et al., 2021), and withdrawal from activities (Clare et al., 2003; Aarsland, 2020; Cao et al., 2023). Up to 90% of people with dementia experience behavioral and psychological symptoms, such as depression, anxiety, apathy, agitation, delusions, sleep disturbances, wandering, and irritability (Bessey and Walaszek, 2019; Lyketsos et al., 2011; Kales et al., 2015; Cao et al., 2023). Moreover, depression not only increases the risk of progression from mild cognitive impairment to dementia but also causes a significant decline in daily functioning and quality of life, increasing the burden on caregivers (Clare et al., 2003; Chan et al., 2020; Watt et al., 2021; Dowson and Schneider, 2021). Dementia is an illness that affects not only the individual, but the entire family. Family members and caregivers are impacted by the challenges that memory problems bring to daily life, as well as the stress and frustration that arise as a result (Clare et al., 2003).

Medications developed to date seem to have the ability to slow down some Alzheimer’s symptom progression (Olazarán et al., 2010), but they come with high costs and undesirable side effects (Maher et al., 2011; Howard et al., 2012; Lin et al., 2015; Abraha et al., 2017; Cao et al., 2023). The limitations of these interventions (Khan et al., 2014; Sykora et al., 2015; Lin et al., 2015; D’Cunha et al., 2019) suggest the need to simultaneously focus on non-pharmacological interventions (Takeda et al., 2012; Irazoki et al., 2020), as they are efficient in improving and maintaining cognitive abilities (Klimova and Maresova, 2017; Wei et al., 2020; Irazoki et al., 2020; Livingston et al., 2020; Xiang and Zhang, 2024) and promoting a good quality of life for both people with dementia and their family (Meyer and O’Keefe, 2020).

### 1.2 Dementia and non-pharmacological interventions

Several non-pharmacological interventions aimed at contrasting the negative effects of cognitive decline that characterized Alzheimer’s and other diagnoses of dementia. Evidence of the brain’s neural plasticity (Mahncke et al., 2006; Nascimento et al., 2021; Takeda et al., 2012; Irazoki et al., 2020) recognizes cognitive training, rehabilitation, and stimulation as effective tools to improve cognitive functions in people with cognitive impairment and dementia (Bahar-Fuchs et al., 2013; Clare et al., 2003).

*Cognitive training* aims to maintain or improve a specific aspect of cognitive function, such as memory or attention (Chan et al., 2020; Ward et al., 2022), through structured and guided practice conducted individually (Davis et al., 2001; Farina et al., 2002; Koltai et al., 2001; Clare et al., 2003; Irazoki et al., 2020) or in groups (Kesslak et al., 1997; Moore et al., 2001; Koltai et al., 2001; Bernhardt et al., 2001; Clare et al., 2003; Bahar-Fuchs et al., 2019; Irazoki et al., 2020). The difficulty level of the activities can be adjusted according to individual functioning (Clare et al., 2003; Irazoki et al., 2020).

*Cognitive rehabilitation* is a personalized approach to optimize physical, psychological, and social functioning (Clare et al., 2003; Bottino et al., 2005; Irazoki et al., 2020; Xiang and Zhang, 2024). The focus is on improving or maintaining cognitive abilities related to daily performance, overcoming deficits, and supporting and enhancing autonomy (Irazoki et al., 2020). As the conditions of people with dementia or Alzheimer’s change, so do the aims of rehabilitation, adapting as impairments become more severe (Clare et al., 2003).

*Cognitive stimulation* is defined as engagement in a range of activities and discussions (usually in a small group) aimed at generally improving cognitive functions, social interactions, and behavior (Clare and Woods, 2004; Cafferata et al., 2021; Oliveira et al., 2021; Gómez-Soria et al., 2023a, 2023b; Xiang and Zhang, 2024). Examples of stimulation techniques include discussion, reminiscence therapy, and reality orientation (Clare et al., 2003; Irazoki et al., 2020; Ward et al., 2022; Chan et al., 2020). These techniques are implemented through a wide range of activities aimed at stimulating thinking and memory, such as discussing past and present events and topics of interest, word games, puzzles, music, and practical creative activities (Gómez-Soria et al., 2023a, 2023b) such as gardening or cooking (Woods et al., 2023; Ryan and Brady, 2023). All cognitive stimulation interventions are typically provided in a group setting (Clare et al., 2003; Irazoki et al., 2020; Xiang and Zhang, 2024; Gómez-Soria et al., 2023a, 2023b; Ryan and Brady, 2023), and they are very useful for older people’s well-being as well. For example, the empowering approach by Vigorelli (2011) can stimulate cognitive functioning and can contribute to the person’s overall well-being. Similarly, dance and aerobic exercise can preserve cognitive activity while simultaneously reducing psychopathological symptoms, like anxiety and depression (Shen and Li, 2016). Music therapy has been shown to reduce depressive symptoms and behavioral issues in people with dementia living in residential care settings, and for those with dementia living in the community, significant benefits come from participating in singing groups (Dowson and Schneider, 2021).

Thus, non-pharmacological interventions seem to foster cognitive functioning as well as psychological well-being in people with a diagnosis of dementia, by representing a very important type of strategy. New actions in this direction can be useful to promote people with dementia quality of life.

### 1.3 Game-based activities as non-pharmacological interventions

A few explored interventions with possible positive effects on cognition and well-being are game-based activities. Play is part of human development (Bruner et al., 1976; Piaget, 1945; Vygotskij, 1934; Winnicott, 1971), and despite it is automatically associated with children (Dell’Angela et al., 2020; Zaharia et al., 2022), several studies have shown that adults (Khan and Pearce, 2015; Kloep et al., 2023) and older people (Estrada-Plana et al., 2021; Chen et al., 2022; Guardabassi et al., 2024) can also benefit from its purpose. There are some interesting results also about people with dementia. The review and meta-analysis by Li et al. (2023) showed that game-based therapy promotes cognitive functioning and reduces depressive symptoms in patients with a diagnosis of dementia. According to the classification proposed by the authors, there are three types of game: *online games* that involve eye-hand coordination (e.g., video games), *interactive somatosensory games* designed for cognitive or motor rehabilitation, and *offline games*, which involve face-to-face interaction between individuals. Several studies have demonstrated the efficacy of the first two types of game: gamification (Sardi et al., 2017), serious games (Contreras-Somoza et al., 2021), and exergames (Astell et al., 2018) have been successfully tested for their potential to facilitate cognitive rehabilitation and enhance the well-being of individuals with Alzheimer’s disease. Instead, there has been a paucity of research examining the potential benefits of offline games for individuals with dementia. Despite the growing evidence that such games can positively impact cognitive abilities (e.g., logical reasoning, memory, and spatial-temporal orientation) and socio-emotional well-being (Vargiu, 2019), few studies have investigated this area. In fact, board games can be identified as a particularly promising avenue of investigation. They involve players placing, moving, or removing pieces on a patterned board (Noda et al., 2019; Chen et al., 2022), making decisions, and interacting with other individuals (Chen et al., 2022). Together, these elements position the game as a potential multi-domain cognitive intervention for individuals experiencing or at risk of cognitive decline (Pozzi et al., 2023).

The majority of the studies conducted in this field have explored the potential benefits of traditional board games for cognitive function and quality of life in older people with dementia. These studies have focused on the game of Go, a form of Chinese chess, and Mahjong, two games very common in Asian countries. Lin et al. (2015) conducted a study with 147 patients diagnosed with Alzheimer’s disease who were unfamiliar with the game of Go. According to the study design, participants were assigned to three conditions: a control group (with no Go game intervention), a short-term Go game intervention group (1 h of gameplay per day), and a long-term Go game intervention group (2 h of gameplay per day). The study lasted for 6 months, and data collected at the end of the study showed that older people who played the Go game had higher cognitive function scores and lower depressive symptoms than the control groups. Similarly, other studies found that Mahjong led to progressive improvements in cognitive performance, including short-term memory, attention, and logical thinking (Cheng et al., 2014; Zhang et al., 2020). Specifically, Cheng et al. (2014) conducted a study to examine the effects of this game on 110 older people with mild-to-moderate dementia who were residents of nursing homes. They were randomly assigned to one of three experimental conditions: a Mahjong group, a Tai Chi group, and a Handicrafts group. In each group, participants performed their activity for 1 h, three times a week, over a period of 12 weeks. The research showed that both Mahjong and Tai chi had a positive effect on delaying cognitive decline, but Mahjong was more effective on short-term memory. Similarly, Zhang et al. (2020) performed a study to investigate the effects of Mahjong in older people with mild cognitive impairment. Participants were randomly assigned to a control group and a Mahjong group. While the control group continued with their daily routine, the Mahjong group was instructed to play in groups of four players in 1-h sessions and to repeat this activity three times a week for a total of 12 weeks. The study showed that playing Mahjong can improve executive function and daily living skills in older people with mild cognitive impairment.

Only a few studies have focused on modern board games and the role they play for older people with dementia. Navarro-Martos and Nieto-Escamez (2022) appear to be the first to have conducted a study with individuals with Alzheimer’s disease and a modern board game. They involved six patients in a cake-building game: half of them had the task of assembling provided pieces with the aim of creating a cake that matched a reference model, while the other half were asked to arrange the pieces in any way they wished. One component of the research team delivered the activity for 3 days a week for 3 weeks, for a total of nine 45-min sessions. Interactions during the sessions were positive for the participants (e.g., they smiled, expressed gratitude, and showed enjoyment). Although global cognitive function remained stable, executive functions showed significant improvement from the initial assessment.

### 1.4 The current study

Based on the importance of non-pharmacological intervention (e.g., Takeda et al., 2012; Irazoki et al., 2020) and board game activities for older people (e.g., Estrada-Plana et al., 2021; Chen et al., 2022; Guardabassi et al., 2024), this study aims to investigate the impact of modern board game activities on people diagnosed with dementia.

The main objective of this study is to determine whether people with dementia can enjoy modern board games and engage with them in a group interaction. Although previous investigations have used one-to-one interactions (Navarro-Martos and Nieto-Escamez, 2022), this study involves participants playing together during their daily activities in nursing homes or day-care centers. This approach is novel in this field of research and can also be valuable from a practical point of view for professionals who work with people with dementia on a daily basis.

Moreover, the purpose of this study is twofold. The first one is to explore the impact of board games on cognitive stimulation, by conducting a new investigation into the short and long-term effects of board games on cognitive functions (e.g., Cheng et al., 2014; Zhang et al., 2020; Navarro-Martos and Nieto-Escamez, 2022). The second aim is to understand their role on psychological well-being by using a biopsychological perspective. Indeed, the present research investigated the role of board games not only in reducing depression symptoms (Lin et al., 2015), but also in promoting well-being in people with dementia.

According to the study’s hypotheses, people with dementia have a positive experience with board game activity, as regularly playing with modern board games stimulates cognitive functioning after a month (H1a), similar to Navarro-Martos and Nieto-Escamez (2022), as well as after 3 months of playing (H1b), consistently with Cheng et al. (2014) and Zhang et al. (2020). In addition, board games can serve as a powerful stimulus for well-being, because in playful activities, people find something that motivates them to play, pleases them, and provides them with satisfaction (Lin et al., 2015; Navarro-Martos and Nieto-Escamez, 2022; Vargiu, 2019). Therefore, it was hypothesized that people with dementia would reduce their depressive symptoms (H2a) and that they would show more indicators of well-being than discomfort while playing board games (H2b).

## 2 Method

### 2.1 Participants and procedure

Participants in the study were older people who had a diagnosis of dementia and attended day-care centers or nursing homes for older people. After obtaining approval from the Ethics Committee of the University of Macerata, the study involved four facilities located in central Italy to accommodate older people: legal representatives provided consent for the older people’s involvement.

Data collection was carried out at the facilities’ headquarters and started with a cognitive screening phase (T0) aimed at determining the MMSE score and participants’ type of diagnosis. People included in the study had an MMSE score between 10 and 24 and a diagnosis of dementia. Older people with Parkinson’s disease were not included in the sample.

Once older people were selected, they participated in an individual session to measure their cognitive functions while the facility’s staff provided data about participants’ level of autonomy and mood (T1). Then, participants were involved in game activities conducted by the facilities’ professionals. Each session lasted approximately 1 h and was carried out twice a week. During the first month, one or two members of the research team took part in the activities once a week to observe participants’ level of well-being and cognitive involvement (S1). At the end of the first month, older people were invited to complete a parallel version of the cognitive tasks used in the first evaluation (T2). For a further 2 months (S2), the professionals in the institutions ran the board game sessions without the participation of the research team. After the second phase (at T3), data were collected on older people’s cognitive functioning and depressive symptoms for the last time. Facilities’ professionals (physiotherapists, psychologists, educators, volunteers) provided their views about social and cognitive aspects of gaming activities.

**Figure 1:**
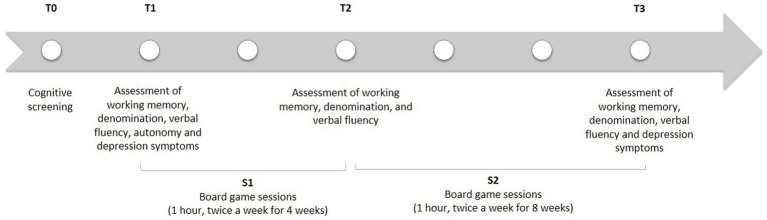
Study design.

Figure 1 shows a description of this study design.

### 2.2 Game activities

Before the study’s implementation, the research team organized a meeting with professionals involved in the research project. All study phases were explained, and a dedicated moment for game sessions was considered. All board games were presented, and the way they should be played was explained. Some of the board games were designed for children; thus, they were adapted in terms of visual appearance, and they were adjusted in terms of difficulty levels to better fit the participants’ abilities. Professionals conducted game activities with a group of older people with a ratio of about 1:5, i.e., one professional and five older people. Participants were gathered around a table, and professionals introduced the board games.

A range of different board games was used to ensure that the participants had content matching their interests and playing dynamics appropriate to their level of abilities.

We used a card game where participants had to mime the movement (such as professions or an object to be used) represented on the card they drew. The operator can mime the movement and ask participants to repeat and guess it or let participants mime the movements and ask the others to guess it.

A puzzle game based on the human body was also used. The operator revealed the puzzle piece, showing it one by one. All together or in pairs, participants had to point to the body part represented on the card to the nearest player or teammate. To increase the difficulty level, the operator placed all puzzle pieces on the table, and, individually or in pairs, participants had to choose puzzle pieces according to the number was drawn and point to body parts represented on cards on their own body.

In another board game, participants have cards representing “letters” and “categories.” Participants had to say a word that begins with the letter drawn or belongs to that category. According to the difficulty level, the operator could place on the table only one of the two types of cards or both together.

Another one consists of choosing one continent and completing it with the cards representing its typical places. The operator drew the cards of the geographical locations one by one and pronounced their names. Participants had to look for them on their boards, and the first player who completed his or her continent had to read all the names on the board.

In a further board game, participants had to roll a die and mime a corresponding movement (eg., 1 = clap your hands) to proceed with the game. To increase the difficulty and stimulate calculation abilities, participants were asked to use two dice with different colored faces. If two numbers with the same colored faces came out, participants had to do an addition. On the contrary, when there were two numbers with different colored faces, they had to do a subtraction and make the corresponding movement.

The last board game required participants to guess the character drawn by the operator by asking questions about the character’s name or physical traits. To increase the level of difficulty, participants could be split into two teams, and each team would draw a character card for the other team to guess.

### 2.3 Measures

#### 2.3.1 Cognitive screening

The Mini-Mental State Examination (Folstein et al., 1975) is a test composed of eight tasks concerning orientation to time, orientation to place, registration, attention and calculation, recall, language, repetition, and constructive praxis. The number of correct answers was summed, and the final score was adjusted according to the criteria by Magni et al. (1996).

#### 2.3.2 Psychological and physical health

At the beginning and at the end of the project, facilities’ professionals filled out a questionnaire regarding participants’ demographic information (T1), autonomy level (T1), and psychological status (T1; T3).

The first part of the questionnaire was about information regarding the participants’ age, sex, type of dementia, any associated comorbidities, and pharmacological treatments. In addition, to evaluate the autonomy level of the participants, the Barthel Index (Mahoney and Barthe, 1965) was used. This observer-based instrument comprises 10 items with three answer options and is aimed at evaluating the level of autonomy in activities of daily living (ADL), including personal hygiene, dressing, toileting, walking, and eating. Responses to each item were weighted according to the scale’s instructions to produce a final index ranging from 0 to 100. The second part of the questionnaire is composed of the Cornell Scale for Depression in Dementia (Alexopoulos et al., 1988), an observational scale composed of 19 items regarding depressive symptoms to which facilities’ professionals answered, ranging from 0 (symptom absent) to 2 (severe symptoms). The scale was translated into Italian by Bianchetti and Trabucchi (2002). Each response was summed, so that lower and higher scores corresponded to low and high levels of depression, respectively. A score higher than 9 on the scale identifies individuals with depressive syndrome. Cronbach’s alpha in this sample corresponds to 0.835 at T1 and to 0.694 at T3.

#### 2.3.3 Cognitive functioning

Cognitive functioning was measured through the Global Examination of Mental State—GEMS (Mondini et al., 2022). Despite the entire instrument being composed of 11 subtasks, for the present investigation, three tasks were selected to assess working memory, verbal fluency, and denomination abilities. As the participants presented poor eyesight, cognitive tasks were selected in which they could verbally answer. Specifically, in the working memory task, the participants had to say the months of the year in the reverse order, starting from December and going back two by two, skipping a month at a time. In the verbal fluency task, the participants had to produce as many words as possible with the letter T (GEMS-A) or D (GEMS-B), under time constraints (60 s). In the denomination abilities task, the participants had to name four color images representing different objects, such as a pear, a table, a saxophone, and a compass (GEMS-A) or a banana, a chair, a harp, and a sharpener (GEMS_B). Each task presents a high correlation with the global score, as GEMS has a high internal consistency. In each task, correct answers (value = 1 point) were summed to have a raw score. Then, for each raw score, a weighted score was computed (each task has a maximum weighted score equal to 9): working memory, verbal fluency, and denomination. In addition, the average score among the weighted scores was computed: verbal functioning. As GEMS has two parallel forms (GEMS-A and GEMS-B), these edits were alternated during the phases of the research project (T0, T1, T2), and they were also alternated among the residential facilities.

#### 2.3.4 Well-being and cognitive functioning during the game activity

During the first period of experimentation (S1), the research team observed game sessions using one of the observation matrices from Quaia’s (2014) text to assess the participants’ well-being. The matrix is composed of two main parts. The first part contains five items concerning the participants’ well-being (e.g., showing interest and attention). The second part contains five items regarding participants’ discomfort (e.g., showing inactivity and passivity). For each of the 10 items, the observer evaluated the behavior of each player as 0 (absent), 1 (rare), 2 (sporadic), 3 (present), or 4 (very present). The mean value of the five items regarding well-being and the five items regarding discomfort were computed to create the well-being and discomfort indices. Each player was observed by one or two members of the research team. Then, the mean value between the two observations was computed. Thus, eight different scales were used: first week well-being index (*α* = 0.867), first week discomfort index (*α* = 0.685), second week well-being index (*α* = 0.826), second week discomfort index (*α* = 0.649), third week well-being index (*α* = 0.830), third week discomfort index (*α* = 0.469), fourth week well-being index (*α* = 0.838), and fourth week discomfort index (*α* = 0.689). Additionally, the 20 well-being items were considered part of the same dimension, the “well-being index” (*α* = 0.945), while the 20 discomfort items were evaluated as “discomfort index” (*α* = 0.825).

In addition, the professionals at the centers completed a questionnaire at the end of the first period of game activities (S1) as well as the second one (S2). It was an *ad hoc* questionnaire used to know their point of view about the experience. Specifically, for this study, professionals were asked to underline important factors to report about cognitive function and well-being.

### 2.4 Data analysis

Data were analyzed using SPSS 24 software. Descriptive analyses, bivariate correlations, and repeated-measures analysis of variance were performed. A content analysis was conducted to determine professionals’ answers to open-ended questions in the intermediate and final questionnaires.

## 3 Results

### 3.1 Participants’ description

According to the study criteria, 36 out of 60 facility guests (*M*_MMSE_ = 18.82; *SD* = 2.96; *Min* = 10.40; *Max* = 22.50) were included as participants. They were 8 men and 28 women aged 86.13 years old (*SD* = 6.94; *Min* = 64; *Max* = 94), and the majority of them have primary school as their educational level (third year of primary school = 2; primary school = 27; middle school = 1; high school = 1; master’s degree = 1). According to the information provided by the facility’s staff, 9 people had vascular dementia and 9 had Alzheimer’s disease, whereas 18 participants had senile dementia. Among the participants, 22 followed a pharmacological treatment for their health conditions (this information was not specified for the other participants). The most common comorbidities were as follows: hypertension (*N* = 6), diabetes (*N* = 6), and anxiety (*N* = 4). Their level of autonomy in everyday life was 64.46 (*SD* = 34.43; *Min* = 10; *Max* = 100).

### 3.2 Cognitive functions

Verbal cognitive functions measured at T1 (*M* = 2.70; *SD* = 0.22), T2 (*M* = 3.27; *SD* = 0.25), and T3 (*M* = 3.06; *SD* = 0.22) did not significantly change over time, *F* (2, 32) = 2.54, *p* = 0.095, *η*^2^ = 0.145. However, as reported in Table 1, analyses showed that verbal cognitive functions significantly changed from T1 to T2 (*p* = 0.029), whereas no differences were found from T2 to T3 (*p* = 0.345) and from T1 to T3 (*p* = 0.196). Specifically, verbal fluency was the only function to show this effect. Despite its measurements not significantly changing from the beginning to the end of the project, *F* (2, 32) = 2.65, *p* = 0.087, *η*^2^ = 0.150, verbal fluency significantly increased after 1 month (*p* = 0.042). Specific details about verbal fluency and the other verbal functions are reported in Table 1.

**Table 1:** Cognitive functions.

Cognitive functions	T1	T2	T3	Δ (T1 − T2)		Δ (T2 − T3)		Δ (T3 − T1)	
	*M* (SD)	*M* (SD)	*M* (SD)	MD	*p*	MD	*p*	MD	*p*
Working memory	1.85 (2.79)	2.47 (2.98)	2.36 (3.34)	−0.619	0.313	0.113	0.842	0.506	0.481
Verbal fluency	1.04 (0.96)	1.51 (1.28)	1.33 (1.20)	−0.478	0.042	0.183	0.493	−0.183	0.493
Denomination	5.20 (2.09)	5.83 (2.12)	5.48 (1.70)	−0.633	0.083	0.352	0.258	−0.352	0.402
Verbal functions	2.70 (0.22)	3.27 (0.25)	3.06 (0.22)	−0.577	0.029	0.216	0.225	−0.216	0.225

Δ, difference; MD, mean difference.

After the first month of activities, two professionals defined the activities as “stimulating,” three of them declared seeing some small improvements (“They seem to be more attentive”), whereas one suggested stability (“Seems to have maintained cognitive function”). One professional emphasized the relation between game difficulty and their involvement: “Residents with better cognitive functioning participated more and there were considerable differences in level among the group.” At the end of the overall experience, four facilities’ professionals emphasized maintenance or slight improvement in cognitive functioning. In particular, one of them revealed that “in general from our observations and evaluations (pre and post) all guests improved or maintained their cognitive level.”

### 3.3 Well-being and depression

The observations conducted during the game activities suggested that the level of well-being (*M* = 2.79; *SD* = 0.52) was significantly higher than the discomfort level (*M* = 0.66; *SD* = 0.32), *F* (1, 35) = 133.69, *p* < 0.001, *η*^2^ = 0.876. As reported in Table 2, differences between well-being and discomfort level were observed in each week of data collection. Furthermore, the results showed that well-being level increased over time, *F* (3, 27) = 7.27, *p* = 0.002, *η*^2^ = 0.510, whereas discomfort level remained stable, *F* (3, 19) = 2.39, *p* = 0.105, *η*^2^ = 0.297.

**Table 2:** Well-being and discomfort indices during board games activities.

First month of activity	Well-being		Discomfort				
	*M*	SD	*M*	SD	*F*	*p*	*η* ^2^
Week 1	2.83	0.73	0.61	0.49	117.99	>0.001	0.792
Week 2	2.79	0.59	0.70	0.51	127.66	>0.001	0.800
Week 3	2.92	0.57	0.56	0.38	183.41	>0.001	0.884
Week 4	3.15	0.62	0.50	0.43	198.61	>0.001	0.880
Overall period	2.79	0.52	0.66	0.32	133.69	>0.001	0.876

In contrast, the initial level of depression reported by professionals (*M* = 2.66; *SD* = 2.85) was not significantly higher than the final ones (*M* = 2.74; *SD* = 2.65), *F* (1, 26) = 0.018, *p* = 0.894, *η*^2^ = 0.001. However, considering the scale as a clinical tool, the results indicated that at the beginning of the project, only one participant presented depressive symptoms (score equal to 14), while at the end of the project, no one had depressive symptoms (the highest score was equal to 9).

At the end of the first month of activities, all facilities’ professionals agreed on the importance of board games to promote well-being. They said that board game sessions “helped to stimulate socialization,” “collaboration,” the creation of “a group identity,” and relationships: board games have “also been useful for joking and in a sense teasing each other” while “improving understanding of each other.” One operator said that “recent arrivals have been observed to have become more familiar with one another, and there is a perception that they have become more integrated into the community of the residence” and that “two guests in particular are one another’s reference points and seek each other outside the game session.” Only one professional reported that guests have taken competitive attitudes and refused to participate in the activity. Group climate was rated positively (“cheerful,” “relaxed,” “serene,” “light”) as “guests are having fun, talking a lot and looking forward to the workshop.” In addition, if “initially the guests were focused on the performance and showed performance anxiety,” then “the atmosphere became more relaxed, guests appear more involved, and they let themselves have more fun.” According to professionals, board games fit well within the facilities’ routines of the various facilities, despite centers that do not have dedicated animation staff present greater difficulties. The activity was enjoyed by older people (“regardless of the degree of participation or the game chosen, all guests report that they enjoy it”) and seems, in particular, to have contributed to increase socialization as guests “group up,” “seek each other out more and seem to recognize each other better even if logically in an implicit way.” Another professional noted “more dialogue,” and a second says she found the participants “more talkative.” In addition to the observed increase in fun and well-being (“better mood tone”), participation in the games also seems to have had a cognitive impact, with participants becoming “more attentive”, and a daily life impact, with participation in games being used as a response to situations and events happening in the facility.

At the end of the 3 months of activities, four professionals answered the final questionnaire and confirmed the same observations: board games helped “stimulate socialization,” “led to cues for dialogue and sharing of memories.” Participants have become more tolerant of each other’s limitations and can maintain good relationships, even after the game experience (“they continue to look for each other outside the workshop”). There was a positive and particularly cheerful atmosphere at playtime. People participated willingly, and the atmosphere was also evaluated positively with respect to the activities conducted in the second and third months of this study. The activity was perceived positively by the operators as the atmosphere was always relaxed, and people were satisfied with the activity. However, one of the operators reports that “the guests remembered the activities and were more bored than previous times, probably the pleasure they experienced playing and the positive emotions associated with the activity left a more important memory trace and therefore more easily retrievable.”

## 4 Discussion

This study investigated the potential of modern board games as a tool that can be adopted in group settings for people with dementia. The results provide interesting insights into cognitive functioning and psychological well-being, suggesting that the role of board games as a possible non-pharmacological intervention should be further explored.

Consistent with previous studies, board games appear to be useful to promote cognitive functions in older people with dementia (e.g., Cheng et al., 2014; Zhang et al., 2020; Navarro-Martos and Nieto-Escamez, 2022). As aforementioned investigations have found positive effects on short-term memory (Cheng et al., 2014) and executive functions (e.g., Zhang et al., 2020; Navarro-Martos and Nieto-Escamez, 2022), this study suggests a possible stimulation in terms of verbal abilities, albeit limited to verbal fluency. It is possible that group interaction, in which verbal exchanges occurred, can support people with dementia in verbalization. Similar to Navarro-Martos and Nieto-Escamez (2022), the role of modern board games was explored, and it was shown that even a less intensive program (two times a week *vs.* three times a week) but spread over time (1 week more) can represent a possible support for cognitive stimulation in older people with dementia. Unlike other research (e.g., Cheng et al., 2014; Zhang et al., 2020), participants in this study showed improvement only after the first phase of the study (i.e., a month), but not after 3 months of experimentation. One possible reason for this is that playing the same board games can become a routine activity, making it less stimulating and less effective from a cognitive point of view. Indeed, people with a diagnosis of dementia have relatively preserved procedural learning (e.g., De Wit et al., 2021; Keith et al., 2023), and they may have developed a familiarity with the activities that make them less motivating (e.g., Hattie et al., 2020; Ryan and Deci, 2020; Csikszentmihalyi, 1990; Ryan et al., 2006). Otherwise, it is also possible that board games become less interesting for the conductors of the activities, and their lower involvement in board games reduces their potential effects. However, from a descriptive point of view, the cognitive performance measured after 3 months was greater than that assessed at the beginning of the project, supporting a possible role for board games in maintaining cognitive functions. Consistent with the results of cognitive tasks, professionals evaluated board games as useful from a cognitive point of view and suggested considering a boredom effect. Thus, by confirming the first hypothesis, the results from this study showed that board games can be useful to stimulate cognitive performance in a short-term period.

Unlike previous investigations (e.g., Lin et al., 2015), this study found that playing board games did not alleviate depressive symptoms in participants. Although previous studies of non-pharmacological interventions have shown an effect after a few weeks of treatment (Gramaglia et al., 2021), these results show that board games are not effective in alleviating depressive symptoms after 3 months, suggesting that these interventions may require a significant amount of time to be effective. Nevertheless, other explanations may also be useful. For example, the low Cronbach’s alpha at T3 suggests that the tool is limited. This may be due to the reduction in participants at the T3 measurement stage, which decreases the already small sample size further, making it challenging to detect a potentially minimal effect. Otherwise, as the majority of participants did not reach the cut-off score, it is possible that the instrument was not sensitive enough for this type of sample, resulting in a floor effect. In this case, a different tool would be more useful.

However, the results suggest that board games can have a positive impact from a health promotion perspective (*vs*. clinical ones). Participants’ state of well-being during the play was higher than their discomfort level. In fact, observed behaviors during board game activities indicate that they can be useful from a psychological point of view. Despite the involvement in board game activities, information collected through first-month observations and professionals’ questionnaire suggested that board game sessions stimulate participants’ well-being. This result is in line with other investigations regarding the role of board games as tools to foster well-being in older people (e.g., Guardabassi et al., 2024; Vargiu, 2019; Bodner et al., 2024), and it is consistent with the psychosocial model of health, which defines health not as an absence of disease, but as the good interaction among physical, psychological, and social dimensions (Engel, 1977). In fact, professionals found board games as an opportunity to stay together, to share experiences and memories, and in some cases, to create a sense of group identity. Consistent with the last hypothesis, board games seemed to be useful to promote well-being in older people with dementia.

These findings expand the literature in different ways with theoretical implications. First, board games can be seriously investigated as a non-pharmacological intervention (Takeda et al., 2012; Irazoki et al., 2020) and extend all this research area that needs to be implemented to address population changes (WHO, 2023). Specifically, to our knowledge, this is the one of the few studies to have explored the role of modern board games and to open up the possibility of reconsidering playing activities differently. A simple board game can be re-designed to be more suitable for people diagnosed with dementia and can be tested to be used for clinical purposes. Future studies can try to combine board games with specific cognitive functions and consider the application of board games also for cognitive or rehabilitation training (Bahar-Fuchs et al., 2013; Clare et al., 2003). Second, the results from this study contribute to health psychology as well, as the role of board games in fostering cognitive functioning and well-being suggests a new area of action that can be used for other clinical groups, for people of different ages, or for healthy older people to promote a good quality of life. Previous studies have suggested that playing has a key role in cognitive, social, and emotional individuals’ life (Bruner et al., 1976; Piaget, 1945; Vygotskij, 1934; Winnicott, 1971) that can lead to a mental state of flow (Csikszentmihalyi, 1990) and reduce depressive symptoms (Lin et al., 2015). Future investigations should clarify all these elements and explore, for example, the role of group composition, the role of each and different board games, and the role of the group leader as possible mediators of the relation between board games and well-being. Third, this study opens up new considerations about aging, specifically about patients with a diagnosis of dementia and their ability to learn. Considering the results of the game activity over the 3 months, where the level of cognitive performance increased in the first phase and remained stable in the following months, it can be said that older people with dementia liked to be challenged by the new activity and were motivated by the games. Within the framework of Vygotskij’s (1934) theory, it can be said that the participants in the study worked in their zone of proximal development in the first part of the activity, whereas in the second phase, after learning how to play, their participation was in the current developmental area. The interpretation of the data collected from this perspective shows that any stage of human life involves developmental and learning tasks. Thus, a new edition of the study can plan to offer a different organization of games over the 3 months, to propose different stimuli, activate the participants’ current developmental area, and proceed with new learning and development.

This study has the quality to have explored the role of board games by collecting information through different instruments (e.g., cognitive tasks, observations, questionnaires) and participants (older people with dementia and facilities’ professionals), a method that is useful to have a comprehensive overview of the phenomenon and to detect the best strategies to promote people’s health (Bishop, 2015). In particular, the inclusion of facility staff offered protection against a hypothesis confirmation bias that can affect the research team who designed the study. In fact, combining data collection with participant observation is an effective way to capture elements and processes within their context and make sense of what emerged through the other data collected (Lanz and Tagliabue, 2020). In addition, the different backgrounds (e.g., psychology, pedagogy, philosophy) of the research team members enrich the results’ lecture and interpretation.

Some limitations of this research project should be underlined as well. The number of participants is small. This reduces the power of the statistical analyses, and it also reduces the generalizability of the results. Additionally, participants have different types of dementia, and their reactions associated with board games can differ due to this difference. Nevertheless, it can be important to understand the behavior and the role of board games in a relatively mixed group, which is very common in nursing homes and day-care centers. This study does not have a control group, which would provide important verification of the intervention’s validity. For this reason, the design of this study suggests that these outcomes should be considered as preliminary results, as future research with a larger number of participants and a control group is needed to better explore the impact of board games on older people with dementia. In addition, it should be noted that the observations were conducted by the research team and by professionals from the facilities, who were aware of the purpose of the study. Although this can represent another limitation, there were two researchers with different backgrounds present for the majority of observations, and professionals from different facilities were involved as experts in their field to express their opinion about the role of board games for their patients. Another limitation is that conductors of board game activities have different professional roles (educators, psychologists, physiotherapists, and volunteers), and their different abilities can affect the board games. To overcome this possibility, a starting meeting was organized with the facilities’ staff, and the first month of observation was also used to ensure the preparation of conductors. All of them showed abilities in the conduction of the board game activities and were able to modify their behavior according to suggestions (e.g., spread out the timing of rounds). Additionally, the nursing home, which involved volunteers as game conductors, organized a training day to increase their knowledge and ability regarding game conduction. A further limitation is that the final questionnaire from professionals has missing responses. This was the case in institutions where the board game conductors had dedicated themselves to the project as an additional activity, which suggests that the research project may also have represented an excess of work for some of them. Beyond the current study, this consideration has implications for the internal organization of institutions for older people, where it is important to have a specific professional dedicated to animation to guarantee the well-being of both the residents and the staff. Their level of well-being and involvement in the project should be taken into account in future studies as a new area of investigation. Finally, this study used different types of board games, reducing the possibility of understanding the most efficient type of game. However, the final questionnaire completed by professionals also collected their observations regarding each game, and this can partially reduce the impact of this limitation.

Nevertheless, current results already offer important considerations for clinical, social, and educational interventions. Professionals should take into account the importance of combining cognitive stimulation and positive emotions. The level of well-being observed during the first month of activities and the increased cognitive performance support the use of board game sessions as an instrument to trigger an individual’s well-being and consolidate a positive memory about the experience. The possible boredom effect can be due to implicit learning (De Wit et al., 2021; Keith et al., 2023), as the emotional component of autobiographical memory is easier to recall than events without emotional valence (Talarico et al., 2004; Schaefer and Philippot, 2005). The positive game experience facilitates cognitive functioning. Thus, combining activities with positive emotions, i.e., valuing people and their skills (e.g., Vigorelli, 2011), should be encouraged. Additionally, to activate the current developmental area in the participants (Vygotskij, 1934), different stimuli and activities should be adopted. Professionals should propose different types of board games as well as alternate board game activities with traditional cognitive stimulation activities. This strategy may be effective in the maintenance of motivation and the stimulation of participation over time. Furthermore, board games can also be used in a family setting to support daily caring activities. They represent an economic solution and have the potential to involve the overall family. For example, as intergenerational activities with older people and children offer promising results for both generations (Gerritzen et al., 2020; Lyndon and Moss, 2023), board game activities should be taken into account as a possible method of intervention capable of promoting well-being in children and in adults with diagnosis of dementia, with a valuable impact from a social and community perspective.

In conclusion, the results of this study suggest that modern board games can be useful in promoting the well-being of older people in nursing homes, and further investigations could provide valuable insights in this regard.

## Data Availability

The raw data supporting the conclusions of this article will be made available by the authors, without undue reservation.
